# Diabetes-induced peripheral neuropathy: Is prescribing physical exercise the answer?

**DOI:** 10.17305/bb.2023.10188

**Published:** 2024-06-01

**Authors:** Saghir Akhtar

**Affiliations:** 1 Division of Human Function and Therapeutics, College of Medicine, QU Health, Qatar University, Doha, Qatar

Diabetes mellitus, a chronic metabolic disorder characterized by hyperglycemia, has become a global health concern with an increasing prevalence worldwide. The International Diabetes Federation (IDF) estimates that over 537 million adults currently have diabetes, and they project that this figure will likely exceed 780 million by 2045 [1]. In addition, a further 541 million adults are thought to exhibit impaired glucose tolerance/pre-diabetes [1]. Among its many complications, diabetic peripheral neuropathy (DPN) affects up to 50% of sufferers [2, 3], with some studies showing that its prevalence, even in pre-diabetes, may be as high as 77% [4]. DPN involves damage to the axons and/or myelin sheaths of peripheral nerves, leading to a range of sensory and motor deficits [3]. The development of DPN is multifactorial, with hyperglycemia serving as a central driver. Chronic hyperglycemia is known to trigger a cascade of detrimental biochemical changes, including the activation of various pathways, such as the polyol pathway, advanced glycation end-product formation, protein kinase C activation, and receptor tyrosine kinases activation. These pathways lead to reactive oxygen species (ROS) formation and subsequent oxidative stress [5–7]. Such hyperglycemia-induced signaling changes lead to a marked increase in ROS, promote endoplasmic reticulum stress, DNA damage, mitochondrial dysfunction, apoptosis, and the activation of proinflammatory signaling. These mechanisms ultimately lead to neuronal injury and dysfunction, including impaired nerve conduction [8]. Furthermore, microvascular complications, which play a crucial role in the development of DPN [2], often result from hyperglycemia-induced endothelial and vascular smooth cell dysfunction. This dysfunction can arise from several factors, including impaired growth factor receptor and nitric oxide (NO) signaling [7]. Diabetes-induced vascular dysfunction leads to impaired blood flow and ischemia in peripheral nerves, contributing to neuronal dysfunction, degeneration and/or loss [8]. Nerve ischemia is especially prevalent in peripheral sensory nerves, leading to reductions in intraepidermal nerve fiber density, especially in those with a longer duration of diabetes [9–11]. Thus, duration of diabetes, poor glycemic control, advanced age, dyslipidemia, hypertension, obesity, and smoking seem to be important risk factors for DPN [3, 8, 12, 13].

As the global prevalence of diabetes continues to rise, along with an aging population, addressing DPN becomes increasingly important in the overall management of diabetes-related complications. DPN often manifests with a spectrum of sensory and motor abnormalities and is typically diagnosed late, by which time irreversible nerve damage may have already occurred [14]. Patients often present with symptoms like tingling, burning sensations, and numbness in the extremities, particularly in the feet and hands (typically referred to as “glove-and-stocking-like” distribution). These sensory deficits can progress to more severe complications, including pain or loss of protective sensation, as well as motor deficits such as muscle weakness and atrophy. This progression further contributes to the functional impairment and reduced quality of life associated with DPN [3]. It is also noteworthy that up to 50% of DPN patients may be asymptomatic, allowing the condition to progress unnoticed [2]. Without early recognition and appropriate preventive measures, DPN patients are at greater risk for injuries, diabetic foot ulcers, and ultimately amputations [2]. Thus, early detection and intervention are paramount in managing DPN and preventing its sequelae [2, 15]. Screening for DPN involves clinical assessments, such as the monofilament test and nerve conduction studies, along with patient-reported symptoms. Currently, there are no effective disease-modifying therapies for DPN that can effectively reverse neuronal damage. Symptom management, such as pain relief, is primarily achieved through pharmacotherapy. However, this approach improves quality of life for some patients but is not effective for all [8, 12, 14, 16–18].

Glycemic control remains a cornerstone in preventing and managing DPN in type 1 diabetes (T1D), emphasizing the importance of comprehensive diabetes care [2, 3, 15]. However, its benefits are modest in individuals with type 2 diabetes (T2D), likely due to the greater prevalence of comorbidities such as obesity and dyslipidaemia, which are independent risk factors for DPN [16, 19]. Contrary to the traditional view that physical activity might be harmful to neuropathic patients with balance and gait issues, prescribing moderate (to even low) levels of physical exercise is now considered to be an important intervention for delaying the onset and progression of both diabetes and DPN [2, 15, 20–29].

In T2D, it is well established that exercise and diet can delay or even reverse the onset of the disease and its complications [2, 15]. However, a recent cross-sectional study conducted in Italy on T1D, which is less studied compared to T2D, suggests that moderate-to-vigorous physical activity might also be a protective factor against developing DPN in T1D (OR ═ 0.221, 95% CI 0.068–0.720; *P* ═ 0.012) [20]. This level of activity was defined as at least 600 metabolic equivalent task (MET) minutes per week, which approximates to about 150 min of moderate-intensity activity or more than 75 min of vigorous-intensity activity per week. In this study, physical activity was assessed using the International Physical Activity Questionnaire among 90 patients. It was found to be low in 21.1% of patients, moderate in 42.2%, and high in 36.7%. They reported a statistically significant reduction in the risk of neuropathy in patients belonging to the group of moderate/high physical activity compared to those with low physical activity [20]. In contrast, a recent UK-based study on patients with T2D suggested that even less than 1.5 h of walking per week, well below the recommended 150 min of brisk walking per week, might be enough to delay the onset of DPN [21]. During a median follow-up of 12.1 years, in 672 individuals (3.7%) diagnosed with DPN, the adjusted hazard ratio (HR) of developing the condition was 0.71 (95% CI 0.53–0.90) for those who engaged in physical activity below the recommended levels compared with those reporting no physical activity [21]. This indicates that some form of physical activity, even at levels that do not significantly impact glycemia, is important for delaying the onset of DPN [30]. It appears that the greatest relative benefits of exercise are achieved at low levels of activity, with additional benefits realized at considerably higher levels of activity than those recommended in prescriptions [27]. A recent meta-analysis supports this, showing that walking at faster speeds lowers the risk of developing T2D, with each 1 km/h increase in walking speed associated with approximately a 9% reduction in risk [31]. Physical exercise prescriptions during pregnancy can also help control gestational diabetes [32, 33], implying that physical exercise programs can be beneficial in managing all forms of diabetes to some extent.

## So how is exercise thought to help in the development of DPN?

Exercise plays a crucial role in managing both diabetes and DPN. Regular physical activity is known to enhance insulin sensitivity, improve glycemic control, reduce body weight, improve blood flow, regulate blood pressure, and enhance overall cardiovascular health. Collectively, these benefits can delay the onset of T2D and its complications [2, 15, 20–29]. Additionally, regular exercise regimens can improve pancreatic β-cell function [34], reverse insulin resistance [35], improve vascular function [28, 36], reduce dyslipidemia and inflammation [25], as well as result in healthier gastrointestinal microflora [37]. These factors may help reduce the risk of developing diabetes and/or improve disease management [23]. For instance, the Diabetes Prevention Program (DPP) trial found that intensive lifestyle intervention, including undertaking at least 150 min of physical activity per week (akin to brisk walking), could prevent T2D by 58% over 3 years [38], with significant benefits still evident after 15 years (27% reduction) [39]. Introducing exercise breaks during prolonged sedentary periods might also be beneficial, as they help lower postprandial glucose levels [15].

Exercise has specific benefits in mitigating the progression and impact of DPN. Regular exercise can promote nerve regeneration, help maintain the integrity of nerve fibers, improve nerve function, and mitigate the progression of peripheral neuropathy [10, 40, 41]. For example, exercise counseling and dietary control in those with impaired glucose tolerance led to the recovery in small nerve fiber function [42]. Additionally, despite the complex relationship between exercise and pain in DPN, several studies suggest that appropriate exercise regimens can help manage neuropathic pain [40–43]. This may be attributed to the release of endorphins, improved circulation, and enhanced neurotrophic support [40–43]. Exercise-induced suppression of pro-inflammatory responses, a reversal in the deficit of NO synthesis/endothelial NO synthase (eNOS) expression, and modulation of voltage-gated calcium channels to affect opioidergic tone in nerves also likely contribute to overcoming peripheral nerve dysfunction and reducing pain [43]. Moreover, as peripheral neuropathy can affect balance and coordination, leading to an increased risk of falls and injuries, exercise programs that include balance and proprioception training, such as Tai Chi, can be beneficial in reducing the risk of falls and improving overall functional capacity [44]. One hour of Tai Chi per session, twice a week for 12 weeks, markedly improved glycemic control, balance, and neuropathic scores in DPN patients [44].

It is important that exercise prescriptions are individualized, tailored to the person’s fitness level, comorbidities, and the severity of neuropathy. This ensures that the chosen activities are safe and appropriate for their specific health status. For DPN patients, a combination of endurance and sensorimotor training is recommended, as these are known to improve insulin action, offer glycemic control, as well as improve fat oxidation and storage in muscle [22, 24, 29]. Regular monitoring of blood sugar levels, foot health, and overall well-being is crucial for individuals with diabetes and DPN who are engaging in physical activity. Any signs of discomfort, pain, or adverse effects should be promptly addressed during careful monitoring programs.

Exercise should always be accompanied by other lifestyle changes, such as personalized dietary prescriptions. A healthy diet can increase lifespan by up to a decade [45]. Since dietary control is almost always recommended alongside physical activity [42], a personalized approach for both diet and exercise (aerobic and resistance) regimens is essential. This approach should undoubtedly include appropriate counseling and regular follow-up to sustain benefits and delay the onset of diabetes and DPN. Recent studies suggest that a healthful plant-based diet is associated with a 24% lower risk of T2D [46], and a low-inflammatory diet is associated with a decreased risk of T2D by as much as 74% [47]. For example, normoglycemic or pre-diabetes subjects on a low-inflammatory diet and with a low genetic predisposition to acquiring T2D, showed a significant reduction (74% [HR ═ 0.26, 95% CI 0.21–0.32] or 51% [HR ═ 0.49, 95% CI 0.40–0.59]) in T2D risk compared to those with high genetic risk on high-inflammatory diets. Thus, a low-inflammatory diet might significantly mitigate the risk posed by genetic factors in T2D development. Moreover, a recent study suggests that epigenetic changes associated with an unhealthy diet, a sedentary lifestyle, and aging may increase the risk of developing T2D and its complications, including DPN [48]. However, whether exercise prescriptions delay diabetes onset or even reverse diabetes and its complications like DPN through normalization of diabetes-associated epigenetic changes remains to be understood.

In conclusion, exercise plays a pivotal role in the management of diabetes and DPN. Tailored and supervised exercise prescriptions, through their impact on glycemic control, neurotrophic support, oxidative stress reduction, anti-inflammatory and anti-apoptotic actions, as well as improved vascular function, offer a comprehensive intervention strategy in the prevention and management of DPN. Furthermore, regular exercise contributes to reversing insulin resistance, reducing weight in obese patients, and improving cardiovascular health, thereby mitigating several risk factors associated with the development and progression of DPN. However, noncompliance with exercise and dietary prescriptions over the long term poses a major challenge to success [26]. Regular, close monitoring, along with counseling and other individualized motivational approaches, might prove beneficial in maximizing the true potential of exercise prescribing for patients with DPN. Further research is needed to establish clear guidelines for exercise prescriptions, taking into account individual variations in DPN presentation and other patient factors such as metabolic state and epigenetics. While exercise is a valuable component of DPN management, it is essential to view it as part of a comprehensive approach that includes glycemic control, lifestyle modifications, and, when appropriate, pharmacological interventions. 
